# Cerebrocentric bias in stroke terminology and the hemovascular paradigm: a conceptual reframing of cerebrovascular disease classification

**DOI:** 10.3389/fneur.2026.1839070

**Published:** 2026-07-27

**Authors:** Maksud Makhmudovich Asadullaev, Nargiza Maqsudovna Vakhabova, Tolibjon Zokhidjon ogli Khomidov

**Affiliations:** Tashkent State Medical University, Tashkent, Uzbekistan

**Keywords:** cerebrocentric bias, cerebrovascular disease, hemovascular paradigm, organ-centered classification, pathogenetic hierarchy, stroke terminology

## Abstract

Stroke remains one of the clinical conditions with the highest global disease burden, yet whose underlying risk factors and pathogenetic mechanisms are increasingly well understood. Nevertheless, the prevailing terminology for stroke—particularly the terms “cerebrovascular” and the broader “cardiovascular”—semantically foregrounds the target-organ manifestation of the disease rather than its etiological center. This article aims to reassess the alignment of stroke terminology with the pathogenetic hierarchy and to analyze the conceptual limitations of organ-centered naming. At the intersection of psycholinguistics, cognitive framing theory, translational stroke research, pan-vascular medicine, and multi-organ disease models, the article proposes the concept of “cerebrocentric bias.” According to this model, the semantic dominance of the organ component in the term “cerebrovascular” may shift clinical reasoning and research priorities from the etiological root toward the phenotypic outcome. The analysis reveals a two-tiered problem with organ-centered terminology: at the macro level, the term “cardiovascular” places the heart at the semantic center, casting stroke into the shadow within the broader spectrum; at the micro level, the term “cerebrovascular” foregrounds the brain, pushing blood composition changes, systemic arterial pathology, and hemodynamic mediation to the background. On this basis, the article advances the hemovascular paradigm as an alternative conceptual framework. This paradigm explains the disease through a three-stage model: the primary stage—blood composition changes and systemic arterial pathology; the secondary stage—hemodynamic disturbances; and the tertiary stage—multi-organ clinical phenotypes. Under this approach, stroke is interpreted not as an isolated event but as the cerebral phenotype of the hemovascular spectrum. The authors regard this proposal not as one that invalidates existing clinical diagnoses but rather as an evolutionary recentering that unifies them etiologically.

## 1 Introduction

Stroke remains one of the most severe epidemiological burdens for the global healthcare system. According to the latest data from the Global Burden of Disease (GBD) 2023 study, in 2023 stroke was the second leading cause of death after ischemic heart disease, claiming 6.79 million lives; disability-adjusted life years (DALYs) lost to stroke reached 157 million (1). The GBD 2021 stroke-specific analysis showed that in 2021, 11.9 million new stroke cases were recorded, 93.8 million people were living with the consequences of stroke, and the DALY figure exceeded 160.5 million (2). The fact that over 87 percent of the global stroke burden falls on low- and middle-income countries demonstrates that this problem carries not only clinical but also systemic and policy significance (3).

Yet against this statistical backdrop lies a deep conceptual paradox. According to the results of the second phase of the INTERSTROKE study, approximately 90.7 percent of stroke cases are directly associated with preventable or modifiable risk factors—namely arterial hypertension, dyslipidemia, smoking, diabetes mellitus, and central obesity (4). Thus, while the causal factors are largely known and modifiable, the global burden of the disease remains remarkably high. In such a situation, the question may lie not solely in the insufficiency of treatment or diagnostic technologies, but also in how stroke is understood, named, and conceptually situated.

The terms “cerebrovascular” and “cardiovascular” have been used in clinical language for over a century, products of the classical nosological model that combines an anatomical organ name with a pathological process. This model emerged in an era when diseases were primarily described through anatomical observation and the logic of localization, and pathogenetic mechanisms had not yet been fully elucidated. However, pathogenetic knowledge subsequently deepened dramatically. Ross (5) defined atherosclerosis not as a local vascular lesion but as a chronic systemic inflammatory disease, demonstrating that it is not limited to lipid accumulation but is governed by an immune-inflammatory cascade. Furchgott and Zawadzki (6) discovered the central role of endothelial dysfunction and nitric oxide (NO) in vascular tone regulation, earning the Nobel Prize. Additionally, the independent significance of hemodynamic-rheological factors such as blood viscosity, erythrocyte aggregation, and fibrinogen in stroke pathogenesis has been confirmed by an increasing body of evidence (7). Yet despite these fundamental discoveries, terminological frameworks have remained virtually unchanged. As a result, today’s clinical language often foregrounds not where the process begins, but where it manifests. The term “cerebrovascular” exemplifies this: it names the cerebral phenotype of a systemic hemovascular process but does not bring vascular wall pathology, hemodynamic mediation, or blood composition changes to the center of the term. It is worth noting that in cardiology, the term “cardiovascular” has over time moved closer to an etiology-based concept: vascular wall remodeling, thrombolytic therapy, and blood rheology management have become established as core treatment strategies, ensuring that the term itself also operates in alignment with etiological logic. In neurology, however, the term “cerebrovascular” has remained unchanged under the influence of neuro-centered traditions, laying the groundwork for the prolonged dominance of the neuroprotection paradigm. The reclassification of cerebrovascular diseases to the nervous system chapter in ICD-11 was an important institutional step (8), yet the terminology itself and its alignment with the pathogenetic hierarchy were not revisited.

In recent years, important initiatives aimed at refining stroke subtypes have emerged—for example, the ISPS25 proposal seeks to revisit ischemic stroke subtypes and phenotypes based on modern diagnostic capabilities (9). However, such initiatives are primarily directed at refining the clinical-subtypic layer, while the terminology itself, its relationship to the pathogenetic hierarchy, and the impact of organ-centered language on clinical reasoning have not been systematically analyzed in the literature. For this reason, the present article is directed not at simply replacing existing nomenclature but at revealing the conceptual mechanism underlying it. It advances the following testable hypothesis: the current organ-centered terminology used to describe stroke does not fully reflect the pathogenetic hierarchy and may therefore act as a conceptual amplifier of an already-existing organ-focused framing in clinical reasoning. We emphasize that terminology is not proposed here as a primary cause of substantive scientific outcomes; rather, it is suggested to operate as one contributing and reinforcing factor among many. Accordingly, if this hypothesis holds, it invites a re-examination not only of how stroke is named, but also of how organ-centered framing may have subtly co-shaped research priorities and the conceptual context within which, for example, the long history of neuroprotective trial failures unfolded—alongside, and secondary to, the well-recognized pharmacological and methodological determinants of those failures.

The primary contribution of this article manifests in three directions. First, the concept of “cerebrocentric bias” is proposed—a conceptual model explaining the relationship between medical terminology, cognitive framing, and clinical decision-making. Second, the two-tiered problem of organ-centered naming is examined within a unified analytical framework: at the macro level, the term “cardiovascular” casts stroke into the shadow within a larger group; at the micro level, the term “cerebrovascular” foregrounds the brain, relegating the pathogenetic root—vascular wall pathology, hemodynamic mediation, and blood composition changes—to conceptual background status. Third, the “hemovascular” approach is proposed as an alternative terminological framework—a conceptual proposal aimed at recentering stroke as the cerebral phenotype of the general vasculohematic spectrum.

Thus, this work views stroke terminology not merely as a linguistic matter but as a theoretical problem with pathobiological, cognitive, and clinical consequences. The subsequent sections analyze the linguistic, biological, and conceptual foundations of this thesis step by step.

## 2 Pathogenetic hierarchy and the limitations of organ-centered terminology

### 2.1 The anatomy of the term “cerebrovascular”

The term “cerebrovascular” is composed of the Latin cerebrum- (brain) and -vascularis (pertaining to vessels) and represents, morphologically, a classical medical compound term that combines an organ name with the localization of a pathological process. The same pattern appears in “cardiovascular,” “renovascular,” and numerous other clinical terms. This naming model has historically relied on identifying a disease not by its etiological root but by the organ in which it manifests. For the era when anatomical observation was paramount, this approach was logically justified. But as modern pathogenetic knowledge has deepened, a fundamental question arises: to what extent does organ-centered terminology reflect the true biological hierarchy of the disease?

From a linguistic perspective, “cerebrovascular” is a neoclassical compound of the X-o-Y type. As Hayashi (10) has shown, in such units morphemes are joined by the linking vowel -o-, and they are often analyzed as “right-headed” constructions; that is, the grammatical category and general superordinate concept are determined by the right component—"vascular.” In this sense, “cerebrovascular” structurally means “a vascular condition pertaining to the brain.” However, the real cognitive processing of compound terms is not determined solely by the grammatical head element. According to the CARIN (Competition Among Relations in Nominals) theory developed by Gagné and Spalding (11), the interpretive relationship in compounds is often activated by the modifier—the first component: the modifier shapes the interpretive frame from the moment the term begins to be read. Thus, in the term “cerebrovascular,” while the semantic referent is formally “vascular,” the initial cognitive dominance may belong to “cerebro-.”

This observation is consistent with experimental psycholinguistic data. Eye-tracking studies by Pollatsek et al. (12) confirmed that complex compound words are processed sequentially from left to right and that first-component properties significantly influence fixation duration and the initial processing stage. From this emerges what may be termed “modifier framing”: a structurally subordinate component exerts cognitively dominant influence.

This modifier framing operates through at least three mechanisms. First, temporal priority: the “cerebro-” component is received before the “-vascular” part is reached, and the initial interpretation is directed toward the brain. Second, informational asymmetry: in the paradigmatic series (cerebrovascular, cardiovascular, renovascular, peripheral vascular), the “vascular” part is the recurring common core, while the distinguishing element is the modifier; consequently, the clinically most “informative” part turns out to be the organ name. Third, clinical specificity: since the vascular system is present in all organs, the diagnostically practical distinction lies in the “which organ?” question—that is, in the modifier.

This terminological problem has been noted previously in the literature. Stehbens (13) analyzed terminology misuse in vascular pathology and emphasized that labeling structures whose nature, cause, or function is unclear with collective terms reduces scientific precision. He assessed “cerebrovascular disease” not as a precise etiological unit but as a “portmanteau term” that groups together diverse vascular lesions in a particular organ. This critique indicates not that the term is incorrect but that it preserves etiological ambiguity at the cost of anatomical-localizational simplification.

According to modern pathobiological understanding, vascular diseases do not “suddenly appear” at the organ surface; they can be understood as at least a three-layered hierarchy. The primary stage encompasses vascular wall pathology: atherosclerosis, endothelial dysfunction, chronic inflammation, oxidative stress. The secondary stage involves hemodynamic and rheological mediation: reduced perfusion, altered flow, increased blood viscosity, microcirculatory disturbance. The tertiary stage represents the organ phenotype: stroke, TIA, vascular dementia, myocardial infarction, ischemic nephropathy, and other target-organ injuries. In this model, the brain is not the starting point; it is one of the clinical manifestations of a systemic hemovascular process. The term “cerebrovascular,” however, places the tertiary stage at the head of the term, leaving the primary pathogenetic layer in the semantic background.

Schmidt (14) interpreted this problem in an even broader context, emphasizing that organ-based compartmentalization is one of the deep methodological limitations of modern medicine. In his view, a single microvascular or endothelial mechanism can simultaneously give rise to several clinical conditions distributed across different specialties—for instance, hypertension, HFpEF, cognitive impairment, and stroke risk—yet organ-specific classification makes it difficult to see this common root. At this point, the problem of the “cerebrovascular” term extends beyond the level of a single word to become an exemplar of the entire epistemology of organ-centered classification.

This can also be observed at the institutional level. The subspecialty of “vascular neurology”—which received formal board certification from the American Board of Psychiatry and Neurology in 2003—in a sense reflects a terminological inversion: neurology is treated as the primary specialty, with “vascular” appended afterward as a modifier. Yet from the perspective of pathogenetic hierarchy, the opposite picture obtains: the vascular process is primary, and the neurological expression is its phenotype. Although Caplan et al. (15) documented the formation of this field as a distinct subspecialty, in this naming as well, the cerebral outcome remains at the center while the etiological root stays on the periphery.

Thus, rather than outright rejection of the term “cerebrovascular,” it is more important to clearly delineate its conceptual limitations. It is useful for expressing clinical localization but does not fully reflect the pathogenetic hierarchy. Precisely this misalignment constitutes the central point for the subsequent sections: if a term places the organ phenotype at its semantic center, how does the common etiological root—atherosclerosis, endothelial dysfunction, hemodynamic mediation, and blood rheology—recede to background status?

### 2.2 Atherosclerosis—the common etiological root

According to modern pathogenetic understanding, atherosclerosis is not a local but a systemic inflammatory process. Ross (5) defined atherosclerosis as a chronic inflammatory disease occurring in the vascular wall, demonstrating that it is not limited to simple lipid accumulation but is governed by an immune-inflammatory cascade involving endothelial cell activation, monocyte–macrophage infiltration, and foam cell formation. Subsequently, Libby et al. (16) characterized atherosclerosis as a “multi-organ phenotype,” emphasizing that the same pathobiological process manifests as different clinical expressions in different anatomical territories. In this sense, myocardial infarction, ischemic stroke, peripheral artery disease, and ischemic nephropathy are not disconnected separate diseases but rather different phenotypes of a common atherothrombotic spectrum.

This concept of unity has been expressed with increasing clarity in recent years through the “pan-vascular medicine” framework. Zhou and Ge (17) established that the human vascular system is a continuous, unified network and argued for understanding atherosclerotic diseases not as separate organ-specific nosologies but as a single pan-vascular condition, proposing pan-vascular medicine as an independent discipline. Wong et al. (2) illustrated this situation with the “blind men and the elephant” metaphor, showing that cardiologists, neurologists, and vascular surgeons may each observe different parts of the same systemic process while losing sight of its unified biological essence (18). This metaphor vividly demonstrates how organ-centered terminology and specialty-based divisions can exert a fragmenting effect on clinical reasoning itself.

Epidemiological evidence likewise supports this unity. In the INTERSTROKE Phase 2 study, approximately 90.7 percent of stroke cases were explained by modifiable vascular and metabolic risk factors (4). By population-attributable risk (PAR), arterial hypertension carried the greatest weight (approximately 47.9 percent), followed by dyslipidemia (26.8 percent), smoking (12.4 percent), diabetes mellitus (3.9 percent), and other metabolic factors. These figures demonstrate that the principal drivers of stroke pathogenesis lie not within the brain but in vascular wall pathology, metabolic imbalance, and systemic hemodynamic disturbances.

Critically, none of these risk factors originates in the brain. Arterial hypertension is related to systemic vascular tone and neurohumoral regulation; dyslipidemia is tied to hepatic lipid metabolism and intestinal absorption; atherosclerotic plaque forms preferentially not in any single organ but in segments of the entire arterial tree where high mechanical stress and flow turbulence are observed—namely the carotid bifurcation, coronary arteries, and peripheral artery branch points. For this reason, interpreting ischemic stroke solely as a “cerebral event” highlights not the pathogenetic center but its organ-level outcome.

Data on polyvascular disease further strengthen this thesis clinically. Tannu et al. (19) demonstrated that approximately 30--70 percent of patients with atherosclerosis have damage in multiple vascular beds, drawing attention to an important paradox: due to organ-based classification and specialty-based divisions, virtually no independent clinical trial dedicated specifically to polyvascular disease has been conducted; existing evidence has been assembled mainly from subgroup analyses of organ-specific trials. This situation illustrates that terminological and classificatory frameworks influence not only how we describe a disease but also which questions we ask at the level of scientific inquiry.

Thus, recognizing atherosclerosis as the common etiological root provides a basis for viewing stroke not as an independent nosological entity but as the cerebral phenotype of a systemic hemovascular spectrum. The discussion here is not about outright rejection of the existing term but about its failure to fully reflect the pathogenetic center. It is precisely this approach that forms the pathobiological foundation of the hemovascular paradigm proposed in this article.

### 2.3 Blood composition, rheology, and hemodynamic mediation

Vascular wall pathology and the systemic atherosclerotic process do not directly lead to clinical events; they are often manifested at the organ level through the flow properties of blood, microcirculatory resistance, and perfusion balance. Blood is a non-Newtonian fluid whose viscosity depends on shear stress; this property is determined by erythrocyte aggregation, deformability, hematocrit, and plasma factors. As Simmonds (20) and colleagues have emphasized, although the flow properties of blood are critically important for tissue perfusion, blood rheology has long remained “the most neglected area” in the study of vascular diseases. For this reason, hemodynamic mediation should be viewed not merely as a “secondary consequence” but as a biological bridge between vascular wall pathology and the organ phenotype. In certain cases, alterations in blood composition and rheological properties turn out to be not the bridge itself but one of the earliest layers of the disease process.

At this point, the classic 1988 observation by Ernst and Matrai (7) assumes particular significance. They compared blood and plasma viscosity, cell filterability, and erythrocyte aggregation in patients with TIA against a control group, demonstrating that impaired blood fluidity may be present not only after a full stroke but also before a clinical cerebral event. The strength of this work lies in showing that rheological change is not merely a consequence of stroke but rather a pre-stroke predispositional layer.

Fisher and Meiselman (21) analyzed hemorheological factors in the context of cerebral ischemia in greater depth, showing that fibrinogen, plasma viscosity, and other hemorheological parameters are associated with stroke severity and the microcirculatory environment. At the same time, they assessed these changes as nonspecific in many cases and noted that they are more prominent particularly in severe stroke. This very nuance is important: hemorheology is not the sole cause of stroke, but it is an independent physiological layer that determines perfusion stability, thrombogenicity, and the fate of ischemic tissue. Other studies also confirm these findings from various clinical perspectives (22, 23).

Recent data show that the clinical significance of this layer persists, though associations differ depending on the endpoint. Thapa et al. (24) analyzed 317 patients who underwent mechanical thrombectomy for large vessel occlusion and found that whole blood viscosity (WBV) values at high and low shear rates were not independently associated with mRS outcome at discharge; the authors noted that this may partly be related to the limitations of formula-based WBV calculation. Such estimating equations derive viscosity from hematocrit and total protein and therefore cannot fully capture the dynamic, non-Newtonian behavior of blood *in vivo*—in particular, the shear-rate-dependent changes in erythrocyte aggregation and deformability that govern viscosity under the markedly different high-shear (large-artery) and low-shear (microcirculatory) conditions encountered in real cerebral perfusion. Consequently, a formula-based value may under-represent the rheological contribution that directly measured, shear-resolved viscometry would reveal. In Noh's (25) study of 298 patients, diastolic blood viscosity was descriptively more closely associated with small vessel disease and cerebral microbleeds, although in multivariable analysis it was not independently associated with stroke subtype or imaging markers; instead, it was more independently associated with hypertension and C-reactive protein. Together, these two studies indicate that blood viscosity is important in stroke biology, but its signal depends on which stage of the process and which clinical timepoint it is measured at.

Endothelial dysfunction constitutes a central component of this mediating stage. Tuttolomondo et al. (26) characterized endothelial dysfunction as one of the key determinants of vascular injury in stroke, noting that it can be detected across different clinical subtypes and, together with inflammatory mediators, affects infarct progression and outcome. The endothelium simultaneously regulates vascular tone, antithrombotic balance, and inflammatory signaling; it therefore serves as the true interface between vascular wall pathology and hemodynamic consequences.

From a hemodynamic perspective, this process is not linear but cyclical. Atherosclerotic changes in the vascular wall disrupt flow velocity, shear stress, and pulsatility; this in turn alters the endothelial response, thrombogenicity, and microcirculatory resistance. Conversely, increased viscosity, enhanced erythrocyte aggregation, and decreased deformability further slow local flow, deepening endothelial stress and wall injury. Thus, the “wall → flow → organ” chain is not unidirectional; it appears far more complete when understood as a “wall ↔ blood ↔ flow” mutually reinforcing cycle.

Viewed from this perspective, the term “cerebrovascular” renders an important layer of the pathogenetic hierarchy invisible within the term itself. In it, the “vascular” component denotes the vessel wall and “cerebro-” indicates the target organ, but blood composition, viscosity, erythrocyte characteristics, and hemodynamic mediation are not named as a separate pathogenetic dimension. Yet existing evidence indicates that this dimension sometimes appears before the clinical event, sometimes correlates with disease severity and background markers, and sometimes influences prognosis. It is precisely for this reason that the “hemo” component is not merely a stylistic addition but serves as a biological basis for recentering stroke as the cerebral phenotype of a systemic hemovascular spectrum.

### 2.4 The “cardiovascular” term: a macro-level terminological problem

In the preceding sections, the problem of the “cerebrovascular” term was analyzed at the micro level: it places the organ phenotype, not the pathogenetic root, at the semantic center. But the limitations of organ-centered naming do not end there. Above it, at the macro level, another large-scale framing exists—the term “cardiovascular diseases.” If the modifier priority demonstrated in Section 2.1 is applied here as well, the “cardio-” component occupies the cognitive center of the entire spectrum, while “vascular” remains as general background. As a result, cardiac pathology becomes not only the clinical but also the semantic and institutional center; stroke, TIA, and other cerebral phenotypes, while formally within this spectrum, are often pushed to the periphery in practical perception.

The historical expression of this phenomenon was described very concisely by Shakir and Norrving: they called stroke a “Cinderella disease,” noting the 62-year “exile” of cerebrovascular diseases within the ICD (8, 27). This phrase is not merely a polemical metaphor; it expresses how stroke was for a long time removed from its natural neurological context and dissolved within the broader “circulatory” framework. This historical process has been analyzed in detail in a separate study Asadullayev et al. (28); the key point for the present article is that stroke’s relegation to the shadows has not only a clinical but also a classificatory and terminological history.

The practical consequences of the macro-level problem are also visible in the numbers. According to GBD 2023 data, in 2023 ischemic heart disease accounted for 8.91 million deaths and 193 million DALYs, while stroke accounted for 6.79 million deaths and 157 million DALYs, making them the two largest components of the global disease burden (1). Thus, while stroke is the close second pillar in terms of scale, the services, organizational models, and policy visibility built around it are not proportionate in many systems. Feigin and colleagues have emphasized that resources allocated for stroke prevention and care remain disproportionately low relative to the disease burden (3). The “blind men and the elephant” metaphor by Wong et al. (18) cited in Section 2.2 also operates at the macro level here: the part of the elephant closest to the heart is perceived as the entire animal.

Thus, the two-tiered problem of organ-centered terminology is clearly visible. At the macro level, the term “cardiovascular” places the heart at the semantic and institutional center, casting stroke into the shadow within the broader spectrum. At the micro level, the term “cerebrovascular” foregrounds the brain, relegating the pathogenetic roots—blood, vascular wall, and hemodynamic mediation—to background status. The problem is therefore not confined to a single word; it pertains to an entire terminological architecture that operates from top to bottom. It is precisely for this reason that in the next section, “cerebrocentric bias” is formulated as a distinct conceptual model.

## 3 Cerebrocentric bias: a conceptual model

### 3.1 Definition

**Figure 1:**
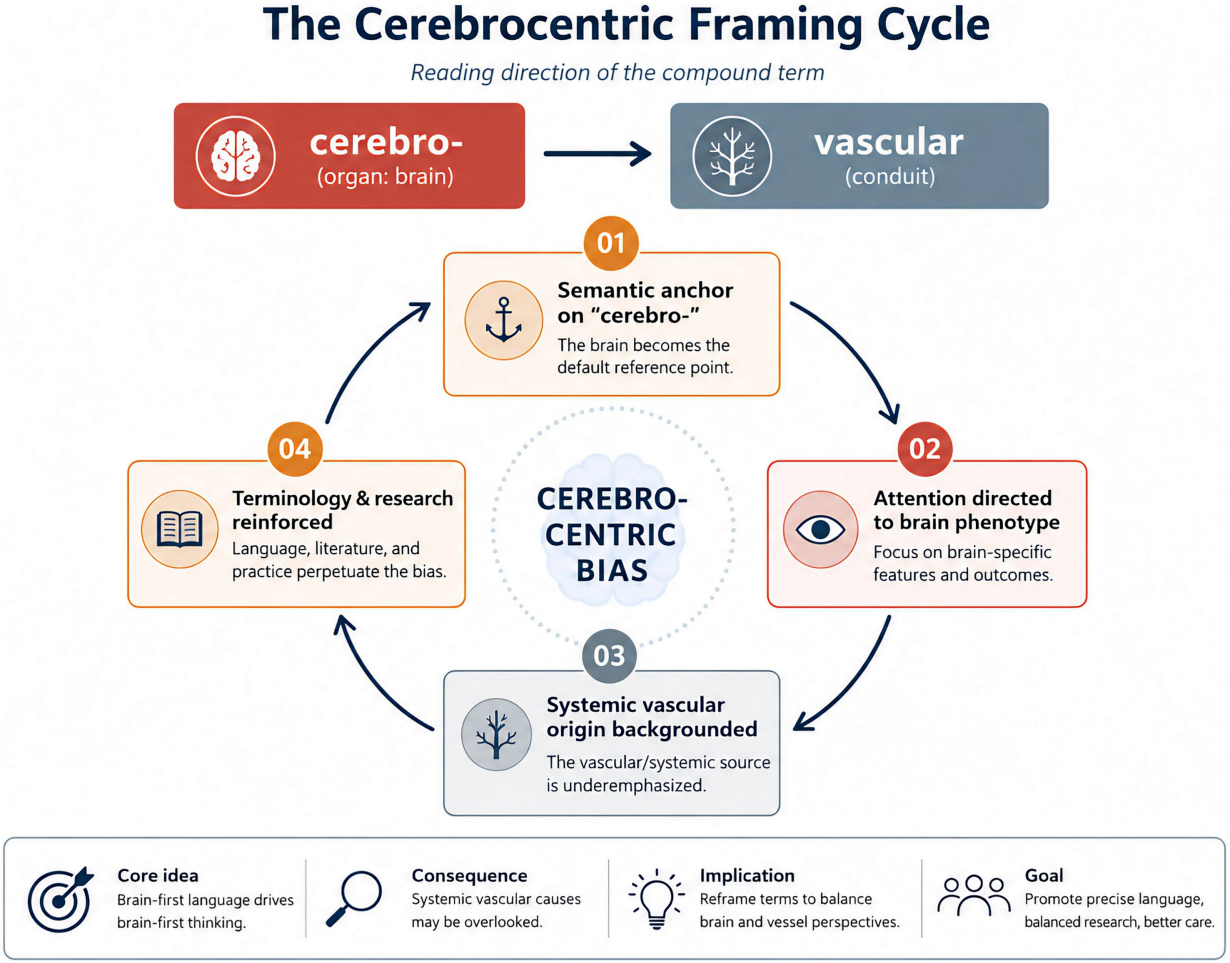
The cerebrocentric framing cycle. Schematic representation of how the organ-centered compound term “cerebro-vascular” reinforces an organ-focused cognitive frame. Because neoclassical compounds are processed sequentially, the leading element “cerebro-” (the organ of manifestation) is semantically foregrounded, while “-vascular” (the conduit) and the broader systemic origin are backgrounded. This semantic anchoring directs clinical attention to the brain phenotype, leaves the systemic vascular origin conceptually peripheral, and—through habitual use in terminology and research—feeds back to reinforce the same framing. The cycle is self-sustaining rather than causal in any single direction.

In this article, the concept of “cerebrocentric bias” is proposed as a conceptual model explaining the relationship between medical terminology, clinical reasoning, and scientific priorities. According to this model, the placement of the organ name at the beginning of the term brings to the semantic center not the etiological root of the pathological process but its target-organ consequence; as a result, vascular wall pathology, blood composition changes, hemodynamic and rheological mediation, and other primary mechanisms are terminologically relegated to the background. Cerebrocentric bias is not a phenomenon that alters biological reality itself; it is viewed as a cognitive-semiotic shift that directs how we see, explain, and determine which layer of the disease we accept as primary. If a disease is called “cerebrovascular,” clinical attention is directed first and foremost to brain damage; even when vascular wall and blood flow balance are acknowledged as causal, they remain not at the center but in the background of the term. Consequently, reasoning is often structured not in the natural direction of pathogenesis but from consequence to cause. Figure 1 illustrates this self-reinforcing framing cycle, in which the leading position of the organ term anchors clinical attention on the phenotype and progressively backgrounds the systemic vascular origin.

This definition rests on the linguistic foundation demonstrated in Section 2.1. There, “cerebrovascular” was analyzed as a neoclassical compound, and it was shown that while formally “vascular” is the superordinate concept, in cognitive processing the first component “cerebro-” may activate the interpretive frame early. The classical work of Taft and Forster (29) on prefixed units, as well as psycholinguistic data on the sequential left-to-right processing of compounds, indicate that the dominance of the first component in perception is not coincidental. From this perspective, cerebrocentric bias is a semantic shift that arises naturally from the internal structure of the term: it makes the “brain” the protagonist of the clinical narrative, leaving the “vascular” component as an accompanying annotation. Here, language and morphology become not merely vehicles of expression but active participants in medical conceptualization.

Cerebrocentric bias may manifest at three interconnected levels: semantic, cognitive, and structural. At the semantic level, the problem lies within the term itself—the organ name is promoted to the head of the term while the etiological center descends to terminological background status. At the cognitive level, this semantic priority may amplify anchoring, framing, and priority errors in clinical decision-making. At the structural level, the same shift is reflected in scientific research questions, service organization, specialty-based divisions, and translational strategies. Thus, cerebrocentric bias is not merely a word-choice problem; it is a multi-layered model that circulates between term, reasoning, and system.

The cognitive-level mechanism can be explained through the general clinical decision-making literature. Croskerry (30) described over thirty forms of cognitive bias in clinical diagnostics and particularly demonstrated how an initial signal can direct subsequent interpretation. Ly et al. (31) showed that the anchoring effect in physician decisions is a real and measurable phenomenon. In the systematic review by Saposnik et al. (32), cognitive biases associated with diagnostic errors were reported to occur in approximately 36.5 to 77 percent of cases across different studies. McNeil et al. (33) demonstrated that presenting the same clinical information using different terms can alter treatment choices by up to 34 percent. These figures do not, of course, lead to the conclusion that they derive directly from the “cerebrovascular” term itself; however, they strongly demonstrate the existence of a general cognitive substrate through which terminological framing can influence clinical reasoning. Warner (34) applied the relationship between language and disease concepts to medicine, emphasizing that how a disease is named directly affects how it is perceived. From this perspective, the expression “cerebrovascular disease” may activate the brain as the central reference point in the clinician’s mind, turning the etiological root into a secondary annotation. Such influence is subtle, often unconscious, and works in conjunction with institutional processes; but precisely for this reason it matters in medical language—terminology often serves as an invisible yet directional frame.

The structural level of cerebrocentric bias is indirectly visible in the translational history of stroke. O’Collins and colleagues documented that over 1,026 neuroprotective agents appeared efficacious in experimental models, yet virtually none of them successfully translated to clinical practice. Gladstone et al. (35) analyzed the failed translational history of over 114 neuroprotective trials. This failure cannot, of course, be explained by terminology alone; methodological, time-window, model-selection, and patient-heterogeneity factors played significant roles. However, this history does show that the prolonged attempt to capture stroke primarily at the endpoint of neuronal damage may not have sufficiently centered the systemic etiological layer. The cerebrocentric bias model is precisely useful for explaining this aspect: when a term places the brain at the center, research too tends to be drawn toward the final outcome in the brain parenchyma.

Against this background, the neurovascular unit concept advanced by Lo, Dalkara, and Moskowitz represented an important turning point: it showed stroke not merely as neuronal injury but as the interaction of neurons, glia, endothelium, and the microcirculatory environment. That the subsequent discussion within STAIR XI also shifted from “neuroprotection” toward “cerebroprotection” is not coincidental—this terminological shift was an attempt to acknowledge that stroke biology encompasses not only neurons but a broader multi-component system (60). Importantly, this evolution does not refute the cerebrocentric bias model; rather, it indirectly confirms it: the very emergence of the need to make corrections at the level of language itself indicates that the previous framework was constraining. Terminological shifts can thus be interpreted as markers of a deeper need for recentering in scientific reasoning.

Thus, cerebrocentric bias is not a simple stylistic problem in stroke naming but a multi-level conceptual model arising from the misalignment of organ-centered terminology with the pathogenetic hierarchy. Its semantic root lies in the modifier framing mechanism described in Section 2.1, its biological foundations in the common atherothrombotic and hemodynamic mediation shown in Sections 2.2—2.3, and its macro-level manifestation in the “cardiovascular” dominance described in Section 2.4. For this reason, this model serves a broader function than a call to rename stroke: by demonstrating the relationship between language, reasoning, and system, it creates a consistent foundation for the theoretical and empirical consequences analyzed in subsequent sections.

### 3.2 Theoretical foundations: language, framing, and cognition

The cerebrocentric bias model is not an idea drawn from thin air; it emerges at the intersection of several independent theoretical strands concerning language, cognition, and the organization of medical knowledge. At the most general level, this model aligns with the weak version—not the strong version—of the Sapir—Whorf hypothesis: language does not absolutely determine thought (71), but it does shape attention directions, categories, and habitual interpretive pathways. In medicine, this is particularly significant because clinical objects are perceived not “ready-made in nature” but through signs, descriptions, categories, and diagnostic names. Warner (34) articulated this idea clearly in the medical context, showing the relationship between language and disease concepts as a central theoretical problem—that is, medical terms not only label an existing condition but also guide how it should be understood and interpreted. From this perspective, the term “cerebrovascular” is not a simple label; it activates a ready-made cognitive frame directed not at the etiological root but at the organ phenotype.

This observation is also reinforced by the sociology of medical discourse. Anspach (36) analyzed clinical case presentations and demonstrated that medical language does not merely transmit information but operates as a “linguistic ritual” that socializes physicians’ ways of thinking. She identified four rhetorical patterns: depersonalization, agentless passive constructions, the presentation of technology as agent, and epistemic markers that subordinate patient narrative to physician knowledge. The significance of this observation for cerebrocentric bias is considerable: if medical language teaches how to see clinical reality, then the internal structure of a term can also influence modes of reasoning. Cassell (37) further emphasized that the purely scientific language of pathology often squeezes out patient experience and the complexity of the process, demonstrating the reductionist tendency of medical language. Cerebrocentric bias can be interpreted as one specific instance of precisely this reductionism: a multi-layered hemovascular process becomes semantically “compressed” around a single organ name.

The next layer of theory leads to the problem of classification and nomenclature. Berlin et al. (38) evaluated the organ-centered structure of the ICD as “static nomenclature relative to clinical needs,” showing a divergence between a purposeful etiological approach and the existing classification system. This critique is important because it elevates the terminology problem from the level of personal speech to the level of institutional architecture. If nomenclature is static, it forces even new biological knowledge into old categories.

Network medicine deepens this critique further. Loscalzo et al. (39), emphasized that in the postgenomic era, classifying diseases solely by organ is preserving excessive reductionism, and they advanced the need to reconceptualize diseases according to their shared biological networks. Subsequently, Barabási et al. (40) developed the diseasome approach, demonstrating that different diseases are connected through shared molecular and cellular nodes. The implication of this approach for cerebrocentric bias is that rigidly delimiting stroke as a “brain disease” does not align with network logic. If the same endothelial, inflammatory, thrombotic, and metabolic mechanisms can produce different phenotypes in the heart, brain, kidney, and peripheral arterial system, then terms built on organ names often reflect phenotypic destinations rather than biological proximities.

Empirical observations on the practical impact of terminology also support this theory. The systematic review by McCulloch et al. (41) showed that medical terminology can influence clinical decision-making, with certain names or technical expressions potentially leading the same condition to be perceived as more invasive or serious. Young et al. (42) demonstrated that medical terms are perceived as “more serious” and more dangerous in the mass media and broader social perception compared to everyday expressions. These studies do not directly examine the term “cerebrovascular,” but they yield one important general conclusion: terms are not neutral; they can alter the intensity of perception, the sense of clinical seriousness, and potentially the direction of decisions.

On this basis, cerebrocentric bias is theoretically explained as follows: language directs attention; medical discourse socializes this direction; institutional nomenclature reinforces it; and organ-centered classification places multi-factorial biology into static categories. This theory does not claim determinism—that is, it does not mean that a term automatically determines every clinical decision. However, it can create a probabilistic cognitive shift and, particularly when combined with historical nomenclature that simplifies a multi-layered etiological reality, may influence clinical and scientific priorities.

### 3.3 Probable translational consequences of organ-centered framing

Explaining the failure of neuroprotective trials solely through terminology would be a scientific oversimplification. In this process, pharmacological limitations, the discrepancy between preclinical models and clinical reality, time-window problems, patient heterogeneity, and translational methodology shortcomings also played significant roles. Nevertheless, this history provides important empirical context for how organ-centered framing may narrow scientific attention. That is, the claim of this section is not that “terminology was the cause” but rather the more cautious conclusion that “the terminological and conceptual framework may have contributed to the shaping of research questions.”

Despite this cautious interpretation, the historical scale itself merits attention. O'Collins et al. (43) analyzed 1,026 experimental neuroprotective agents, of which 114 were taken to human clinical trials, yet virtually none achieved sustained clinical success. Gladstone et al. (35) and colleagues noted in their analysis of these translational failures that attention was often focused on preserving cerebral gray matter, while white matter, endothelium, glial cells, and the broader microcirculatory environment remained relatively sidelined. In this sense, the historical “neuroprotection” paradigm adhered more strongly to the final parenchymatous outcome of the pathological process, while the systemic and vascular layers often remained in the background. As early as the NINDS Stroke Progress Review Group discussions (2001) (64), the need to broaden translational strategies and view stroke as a unity of several cellular and vascular components began to be felt.

Importantly, the field itself began to sense this constraint over time. When Lo, et al. (44) developed the neurovascular unit concept, they expanded the research object from the neuronal level to the level of the entire cerebral microenvironment. Zhang et al. (45) proposed a shift from “neurovascular unit” toward “vascular-neural network,” demonstrating that stroke biology should be understood not as a static single unit but as a multi-nodal and multidirectional network. Tiedt et al. (46) further broadened this view, characterizing stroke not merely as a local cerebral event but as a process connected to systemic biology, peripheral immune response, and multi-organ adaptations. Thus, the scientific evolution within the field has proceeded in one direction: neuroprotection → neurovascular unit → vascular-neural network → systemic biology. This evolution is linked to the recognition that earlier, narrower models were insufficient.

It is precisely here that the terminological question becomes pertinent once more. If the field itself has been progressively moving away from neuron-centered and organ-centered conceptualizations toward increasingly vascular, network-based, and systemic models, then the historical naming and initial conceptual framing were, at the very least, narrow to some degree. Of course, this narrowness alone cannot explain the failure of 1,026 agents. But it is not inconceivable that it influenced which targets were deemed “primary,” which components “supplementary,” and which biomarkers “secondary.” For this reason, interpreting the history of neuroprotection not as direct proof of cerebrocentric bias but as an illustrative empirical context demonstrating the probable translational consequences of organ-centered framing is more appropriate.

### 3.4 Clinical and research consequences

Cerebrocentric bias does not remain merely a theoretical model; it entails consequences that may indirectly yet systematically affect clinical practice, research priorities, and healthcare policy. The claim here is not deterministic: it is not concluded that every clinical decision or every scientific direction is directly determined by terminology. However, how a disease is named and which layer is placed at the semantic center can typically influence which questions are considered primary, which mechanisms secondary, and which monitoring modalities peripheral. From this perspective, at least five significant consequences of cerebrocentric bias can be identified.

The first consequence is semantic influence on physician reasoning. The term “cerebrovascular” directs physician attention toward the brain—toward the target-organ and outcome stage of the disease. As a result, CT, MRI, perfusion imaging, and the degree of neurological deficit take center stage in the diagnostic algorithm, while vascular wall pathology, blood composition changes, endothelial status, and the rheological environment may be pushed to the background. This mechanism does not remain a theoretical conjecture: the observation by Ly et al. (31) showed that the initial diagnostic label in triage documentation can significantly influence the scope of subsequent investigations. Thus, it is not inconceivable that how a disease is initially placed within a semantic frame also directs the subsequent clinical roadmap. The problem here lies not in physician knowledge but in the early cognitive influence of terminological framing. Von Kummer and Babinec (47) also emphasized that certain expressions in stroke medicine language can be ambiguous or misleading, showing that inaccurate or vague expression is not merely a semantic problem; it can also affect patient selection, interpretation of imaging findings, and evaluation of treatment options.

The second consequence is the prolonged dominance of the neuroprotection paradigm. If stroke is conceptualized primarily as a “cerebral event,” therapeutic and scientific strategy naturally gravitates toward saving the neuron. As shown in Section 3.3, in the translational failure history of 1,026 experimental agents and 114 clinical trials, attention was often concentrated on the final parenchymatous outcome of stroke, while mediating layers such as the vascular wall, endothelium, and microcirculation received relatively less focus. The subsequent evolution within the field—from neuroprotection to cerebroprotection, and from there to the neurovascular unit and systemic biology—indicates an internal recognition of this constraint. Yet everyday clinical language has continued to preserve the organ-centered framework.

The third consequence is the weak development of endothelial and rheological monitoring. In modern stroke biology, endothelial dysfunction, thrombogenicity, microcirculatory resistance, and rheological properties of blood are among the central mediating layers. Tuttolomondo and colleagues characterized endothelial dysfunction as one of the fundamental determinants across different clinical subtypes of stroke. Classical works by Ernst and Matrai, Fisher and Meiselman, as well as studies, have shown that hemorheological disturbances may carry clinical significance. Nevertheless, systematic assessment of blood viscosity, fibrinogen, erythrocyte deformability, or endothelial function is not yet a central part of the routine stroke algorithm in many centers. The reason for this disproportionality may not solely be a matter of resources or standards; it may also be related to the conceptual secondary status of the etiological stage. If the term constantly redirects toward the brain outcome, mediating pathogenetic layers can more easily fall from monitoring priorities.

The fourth consequence is the weak development of multidisciplinary integration. The term “cerebrovascular” places stroke primarily within neurology, whereas its etiological roots often intersect with cardiology, angiology, hematology, endocrinology, and nephrology. Hachinski et al. (48) demonstrated the shared vascular basis of stroke, vascular cognitive impairment, and dementia, emphasizing the need for an integrated approach. The pan-vascular medicine direction points in exactly the same direction: atherosclerosis and endothelial dysfunction are not separate organ diseases but the common root of a multi-organ spectrum. If a single biological mechanism can produce myocardial infarction in the heart, stroke in the brain, and ischemic nephropathy in the kidney, then rigid boundaries between specialties are more historical and institutional in character. Organ-centered terminology indirectly reinforces these boundaries, potentially slowing recognition of the common etiological platform.

The fifth consequence involves lessons from the “brain attack” campaign. This concept, advanced by Hachinski (49), aimed to reimagine stroke as an urgent, time-sensitive, and preventable condition analogous to “heart attack.” This initiative was practically effective: the stroke center model, emergency response logic, and the “time is brain” paradigm significantly influenced clinical organization. However, the limitation of this experience is also clear: rather than dismantling the organ-centered framework, it to some extent reinforced it—the expression “brain attack” frames stroke not from the perspective of vascular wall and hemodynamic mediation but as an “attack” directed at the brain. Thus, this campaign established urgency but could not fully reconstruct etiological centering. Terminological reform must account not only for communicative effectiveness but also for alignment with the pathogenetic hierarchy.

In this way, cerebrocentric bias manifests as a conceptual effect with potential visibility in five clinical and scientific directions: it semantically influences physician reasoning, may tilt research attention toward the final neuronal outcome, pushes endothelial and rheological monitoring to the periphery, slows multi-network integration, and may impede full utilization of terminological reform experience. This model should be viewed not as an inevitable cause but as a theoretical mechanism explaining the misalignment between the pathogenetic center and the organ-centered clinical framework. It is precisely this misalignment that grounds the hemovascular paradigm proposed in the next section not merely as a linguistic novelty but as a conceptual necessity.

## 4 The hemovascular paradigm

### 4.1 Conceptual model

The hemovascular paradigm proposed in this article envisions restructuring the pathogenetic hierarchy based not on the organ but on the etiological center. In this model, the initial identifier is not a particular target organ but systemic arterial pathology together with the blood composition and rheological changes that shape it; organs are interpreted as clinical phenotypes of this common process. Therefore, the hemovascular paradigm does not simply negate the concept of “cerebrovascular” but places it within a broader pathobiological context: stroke is not an independent and isolated disease but one of the cerebral manifestations of the systemic hemovascular spectrum. This paradigm accomplishes more than proposing a new term---it brings to the semantic center not where the disease manifests but which mechanism underlies its formation.

This proposal is formed at the intersection of at least four independent strands: pan-vascular medicine, network medicine, multi-organ syndrome approaches, and tissue-agnostic oncology. At the clinical level, this shift is already being observed. In the COMPASS trial, 27,395 patients with coronary and peripheral artery disease were united as a single atherothrombotic platform (56), and the combination of rivaroxaban and aspirin was shown to reduce major vascular events; polyvascular patients derived the greatest absolute benefit. The CANTOS trial powerfully confirmed the systemic inflammatory nature of atherosclerosis: canakinumab reduced cardiovascular events without directly affecting lipid levels. The shared lesson of these two trials is that clinical benefit derives not from an organ-separated approach but from targeting the etiological center.

At the heart of the hemovascular paradigm lies a three-stage pathogenetic model. The primary stage encompasses blood composition changes and systemic arterial pathology; the secondary stage involves hemodynamic disturbances; and the tertiary stage comprises multi-organ clinical phenotypes. A key innovation of this model is that blood composition and rheological properties are no longer viewed as mere “additional factors” of vascular wall pathology but as co-equal components of the primary stage. The “hemo” component is not a stylistic element added to the model later but is introduced as the etiological centering itself.

The primary stage constitutes the true etiological core of the disease. Here, two parallel but mutually reinforcing processes occur. On one hand, blood composition changes—the retention of apoB-containing lipoproteins in the vessel wall, increased blood viscosity, erythrocyte aggregation, decreased deformability, and activation of inflammatory mediators—create the earliest foundation of the atherothrombotic process. The “response-to-retention” hypothesis developed by Williams and Tabas (50) identifies lipoprotein retention in the vascular wall as the necessary central event of early atherogenesis. The experiment by Schwenke and Carew (51) demonstrated that LDL accumulation occurs well before fatty streaks and visible lesions appear, revealing the temporal priority of blood composition changes. Ernst and Matrai’s classic observation showing rheological disturbances prior to TIA, as well as data from the Edinburgh Artery Study showing that baseline blood viscosity is a strong predictor of future vascular events, support precisely this stage (59).

The second side of the primary stage is systemic arterial pathology—atherosclerosis, endothelial dysfunction, oxidative stress, and a tendency toward thrombogenicity. Crucially, blood composition changes and vascular wall pathology do not operate as two separate lines but as a cyclical and mutually reinforcing system: blood composition disturbances amplify wall pathology, and inflammation and dysfunction in the wall in turn further worsen the rheological environment. For this reason, the hemovascular paradigm does not separate these two dimensions; rather, it views them as two co-central components of the primary stage. The placement of the “hemo” part first in the term “hemovascular” semantically reflects precisely this sequence.

The secondary stage—hemodynamic disturbances—is the functional expression of the primary pathogenetic process. At this stage, flow velocity and direction change, perfusion pressure imbalance occurs, microcirculation is disrupted, and favorable conditions for embolism or thrombosis are created. It is precisely this stage that serves as the “functional bridge” between the etiological center and the clinical event. This link is important because it demonstrates that stroke is not an event that “suddenly appears in the brain”; rather, it is often the acute clinical expression of an already-formed hemovascular imbalance in a particular vascular bed. In this way, the hemodynamic stage mechanistically completes the model: blood and wall pathology reach the clinical phenotype not directly but through perfusion-hemodynamic mediation.

The tertiary stage—multi-organ phenotypes—represents the various anatomical expressions of the common hemovascular process: cerebral phenotypes (stroke, TIA, vascular dementia), coronary phenotypes (myocardial infarction), renal phenotypes (ischemic nephropathy), and peripheral phenotypes (peripheral artery disease). They are understood not as separate independent diseases but as organ-specific manifestations of a common spectrum. The high risk in polyvascular patients shown by Tannu et al. (see Section 2.2) supports precisely this spectrum approach. Table 1 reflects this multi-organ spectrum.

**Table 1:** Conceptual comparison of the Cerebrovascular and Hemovascular paradigms.

Feature	Cerebrovascular paradigm (Current)	Hemovascular paradigm (Proposed)
Focus and cognitive anchor	Brain tissue, neuronal structures (Consequence)	Blood flow, rheology, and arterial wall (Cause)
Pathogenetic hierarchy	Inverted: Consequence placed first	Correct: Etiology placed first
Therapeutic target	Neuroprotection/Cerebroprotection	Rheological balance, endothelial protection, recanalization
Clinical approach	Organ-centered (Nervous system only)	Systemic/Pan-vascular (Multi-organ phenotype integration)
Research vector	Directed at brain ischemia and neurological deficit	Directed at inflammation, coagulation, and vascular biomechanics

This model rests on four core principles. First, etiological centering: classification is built on the pathogenetic root rather than the organ. This approach has an analogy with tissue-agnostic therapies in oncology: the approval of pembrolizumab for solid tumors with a specific genetic feature, regardless of organ of origin, was a strong signal of the transition from organ-based to molecular-marker-based taxonomy. Second, the spectrum approach: clinical events are viewed not as separate entities but as phenotypes of a common process. This resonates with the CKM syndrome proposed by the AHA in 2023 (65), which views cardiovascular, kidney, and metabolic disturbances as a single systemic platform. Third, preventive priority: if the etiological center lies in the primary stage, therapeutic attention shifts there as well. Here, the INTERSTROKE paradox, CANTOS results (66), and general atherothrombotic biology complement one another: targeting modifiable risk factors and the inflammatory component is an attempt to manage the root, not the organ phenotype. Fourth, multidisciplinary integration: the hemovascular paradigm brings neurology, cardiology, angiology, hematology, and nephrology together on a single etiological platform. Hachinski and colleagues’ call for an integrated vascular approach, as well as the ESC’s 2024 unified guidelines on peripheral arterial and aortic diseases (62), indicate that institutional movement in this direction has already begun. The hemovascular paradigm unites these fragments within a single theoretical model.

The term “hemovascular” itself already exists in the literature: Cap (52) used it in the context of “hemovascular dysfunction,” Hu (53) in the form of “hemovascular progenitors,” Miyazaki in the context of “hemovascular niche,” and Aird (54) in the framework of a “hemovascular system” concept. Thus, the proposed term is not an artificially created empty construct; it is intuitive and morphologically comprehensible for scientific language. The novelty of the present article lies not in the term itself but in applying it at the paradigm level for stroke and vascular disease terminology. For this reason, the word “hemovascular” is proposed here not as new jargon but as a term with an already-existing scientific root yet a new conceptual function. The morphological structure of the term is itself significant. In the neoclassical compound “cerebro-vascular,” the leading element names the target organ and the following element names the conduit, so that the sequence places the phenotypic destination first and the vessel second. “Hemo-vascular” inverts this priority in a manner that mirrors the pathogenetic hierarchy: the leading element “hemo-” denotes the blood content—the compositional and rheological substrate in which the earliest disease process resides—while “vascular” denotes the conduit through which that substrate acts. In morphosyntactic terms, the modifier thus shifts from the organ of manifestation to the etiological medium, placing semantic emphasis on the circulating cause rather than the downstream target. This is precisely why “hemovascular” is not merely an alternative label but a morphologically motivated reordering: the order of the constituents encodes the order of the pathogenesis.

**Figure 2:**
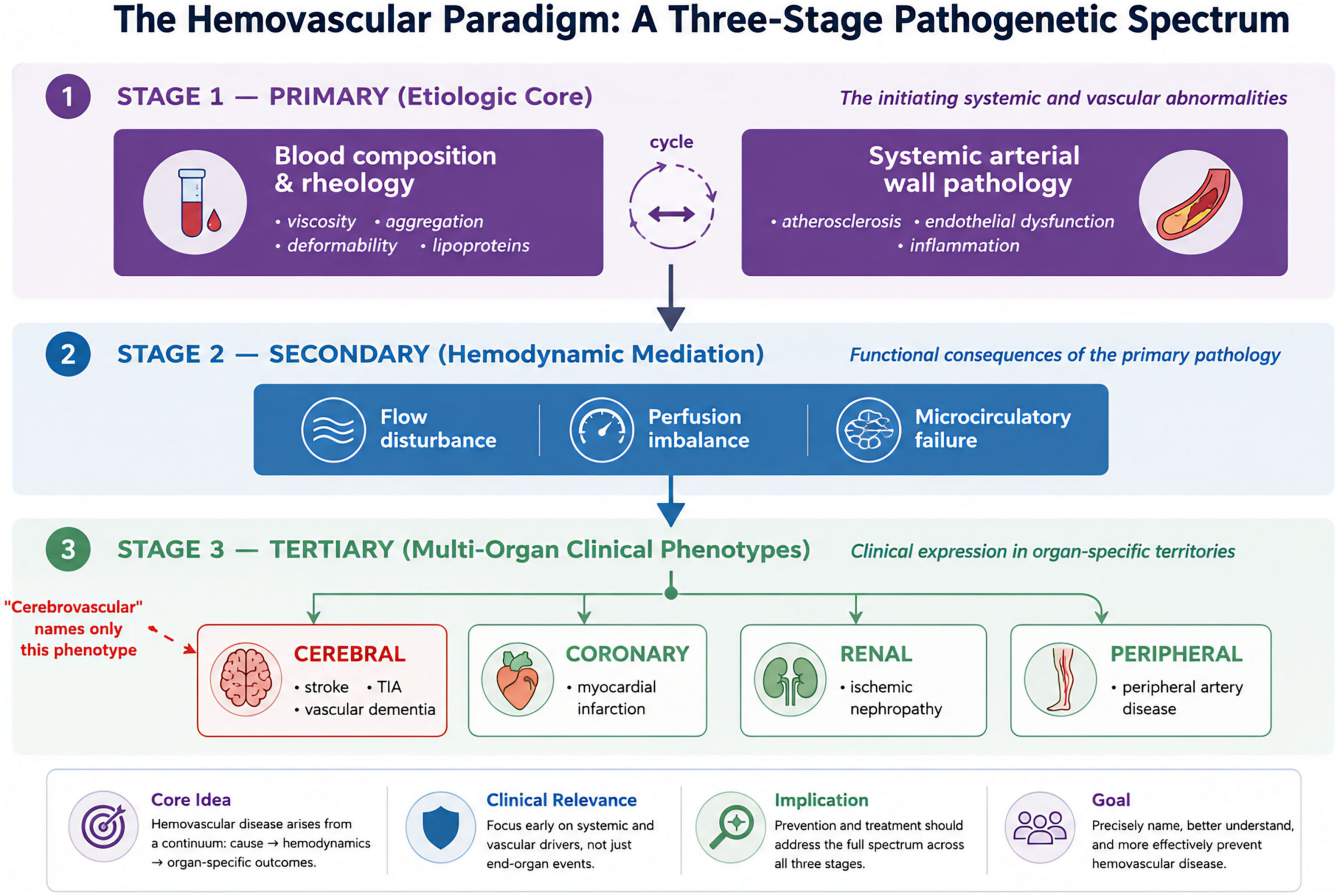
The hemovascular paradigm: a three-stage pathogenetic spectrum. The model re-centers classification on the etiological core rather than the organ of manifestation. Stage 1 (primary, etiologic core) comprises two co-equal and mutually reinforcing components—blood composition and rheology, and systemic arterial wall pathology. Stage 2 (secondary) is hemodynamic mediation, the functional bridge between the etiological core and the clinical event. Stage 3 (tertiary) comprises the multi-organ clinical phenotypes—cerebral, coronary, renal, and peripheral—understood as organ-specific manifestations of one shared process. The conventional term “cerebrovascular” names only the cerebral phenotype (highlighted), whereas the hemovascular lens encompasses the full spectrum and foregrounds its common origin.

Thus, the hemovascular paradigm does not simply call for replacing the term “cerebrovascular”; it proposes restoring the pathogenetic hierarchy. Its internal logic is clear: blood composition changes and arterial pathology → hemodynamic disturbances → multi-organ phenotypes (Figure 2). Within this hierarchy, “cerebrovascular disease” appears not as an independent singular category but as the cerebral component of the hemovascular spectrum. It is precisely this recentering that constitutes the core theoretical proposal of this article.

### 4.2 Historical and terminological precedents

The hemovascular paradigm is not an entirely unprecedented proposal. In the history of medicine, the renaming or reconceptualization of diseases has occurred several times, particularly when an existing term reflected the biological mechanism in a narrow, imprecise, or misleading manner. Importantly, such changes have typically been carried out not to “find a new word” but to bring clinical and scientific understanding closer to the etiological center. From this perspective, the four most important precedents for the hemovascular proposal are as follows.

The first precedent is the transition from NAFLD to MASLD (67). This is perhaps the methodologically closest analogy. In 2023, through a multi-society Delphi consensus process, the name itself was examined as a problem: 74 percent of participants indicated that the existing NAFLD/NASH nomenclature was sufficiently flawed and that the name should be changed; the predetermined supermajority threshold for consensus was 67 percent. In the final selection, 75 percent of the external expert panel endorsed MASLD over NAFLD. The essence of this change is critically important: the primary semantic emphasis was shifted away from external, negative, or stigmatizing descriptors such as “non-alcoholic” and “fatty” and onto the central pathogenetic mechanism—metabolic dysfunction.

The second precedent is tissue-agnostic oncology. This represents the most radical paradigm shift, as it demotes organ or tissue origin from primary identifier to secondary status. In 2017, the FDA granted pembrolizumab the first tissue-agnostic approval for MSI-H/dMMR solid tumors; in 2018 (55, 69, 70), larotrectinib was approved for NTRK fusion-positive tumors under the same paradigm. The significance of this precedent is that it brings to the center of clinical classification not the question “in which organ?” but rather “on the basis of which mechanism or biomarker?” The hemovascular paradigm proposes an analogous—but domain-specific—shift for vascular diseases.

The third precedent is IgG4-related disease (IgG4-RD) (68). This is structurally one of the closest analogies, as it unified conditions previously regarded as separate organ diseases under a single fibro-inflammatory mechanism. The systemic nature of IgG4-RD was initially recognized through pancreatic forms and subsequently its multi-organ character became apparent. The 2011 international pathological consensus and 2012 comprehensive diagnostic criteria consolidated this unification. As a result, previously separate pancreatic, biliary, renal, and other manifestations began to be understood under a unified mechanism-based diagnosis. This provides an important lesson for the hemovascular paradigm: sometimes it is possible—and scientifically and clinically more useful—to reorganize multiple organ phenotypes under a common etiological platform while preserving them.

The fourth precedent is CKD-MBD. In 2006, KDIGO recommended restricting the term “renal osteodystrophy” to bone morphological changes only and introducing the broader systemic syndrome term CKD-MBD (63). Importantly, this new entity encompassed not just bone changes but mineral metabolism disturbances, bone pathology, and vascular or other soft-tissue calcification as a unified syndrome. This too resonates deeply with the hemovascular proposal: in both cases, a narrower, historical term does not adequately reflect the broader systemic nature of the disease.

Together, these four precedents yield one general conclusion. In the history of medicine, nomenclature reform typically occurs under three circumstances: first, when an old term conceals the biological mechanism; second, when a common systemic process is identified behind separate organ phenotypes; and third, when a new term can better direct clinical decisions and research priorities. MASLD, tissue-agnostic oncology, IgG4-RD, and CKD-MBD each exemplify different combinations of these three criteria. Accordingly, the “hemovascular” proposal is neither terminologically unusual nor historically unfounded; rather, it is a consistent continuation, in the domain of vascular diseases, of the logic of etiological recentering already observed multiple times in modern medicine.

### 4.3 What the paradigm changes and what it does not change

The hemovascular paradigm does not propose to abolish existing organ-centered clinical diagnoses. It does not deny the clinical significance of “stroke,” “TIA,” “myocardial infarction,” “peripheral artery disease,” or other phenotypic entities; rather, while preserving them, it proposes a higher-order framework that unifies them etiologically. In other words, this paradigm does not eliminate phenotypic diagnoses but repositions them within a common pathogenetic spectrum.

For this reason, the aim of the hemovascular approach is not to abruptly and entirely replace the term “cerebrovascular” but to shift the center of clinical reasoning from the target organ to the etiological root. In this model, stroke is interpreted not as an isolated event but as a hemovascular cerebral phenotype. Likewise, coronary, renal, and peripheral manifestations are understood as other phenotypes of the common hemovascular spectrum. Thus, this paradigm does not diminish diagnostic precision; on the contrary, it makes visible the common biological logic behind separate clinical entities.

## 5 Discussion

### 5.1 Counterarguments and responses

The proposed hemovascular paradigm naturally invites a number of objections. Addressing these objections proactively is important, as the aim of this article is not to polemically negate existing terminology but to demonstrate its conceptual limitations and propose an alternative framework.

The first counterargument: the term “cerebrovascular” is already adequate; it contains both a “cerebral” and a “vascular” component. At first glance, this objection appears reasonable. However, the problem lies not in the presence of the components but in their semantic order and conceptual center. As demonstrated in Section 2.1, in compound terms the first component often holds cognitive priority; thus, the “cerebro-” head draws attention toward the site of consequence, while “-vascular” remains at the level of explanatory background. The difference between “cerebrovascular” and “hemovascular” is therefore not merely stylistic but hierarchical: the former foregrounds the organ phenotype, the latter the etiological center.

The second counterargument: ICD-11 has already resolved the problem; cerebrovascular diseases have been returned to the nervous system chapter. This objection is partially valid. ICD-11 took an important step: returning cerebrovascular diseases to the nervous system chapter was a significant historical and structural correction. However, vascular dementia still resides within neurocognitive disorders; the term “cerebrovascular” itself has not changed; and the full integration of the spectrum has not been completed. As will be analyzed in detail in subsequent articles, the ICD-11 architecture does not eliminate all cross-chapter fragmentation. Therefore, while ICD-11 represents a structural step forward, it cannot be said to have definitively resolved the terminological and conceptual issue. At the same time, new etiological-subtypic systems such as ISPS25 serve to more precisely differentiate phenotypes within stroke, but the terminology itself---namely organ-centered naming and its alignment with the pathogenetic hierarchy---remains a separate-level problem. ISPS25 and the hemovascular paradigm do not negate each other; one addresses internal phenotyping, the other a higher-level conceptual framework.

The third counterargument: terminology does not have a real impact on clinical practice. This objection is important, and the response must be measured. Clinical practice is not governed by words alone; evidence, guidelines, resources, and institutions also shape it. However, systematic reviews show that naming the same condition differently can influence management preferences and psychological perception: more “medicalized” or more precise terms have been associated with more invasive management and higher risk perception. Historical nomenclature reforms in medicine also indirectly confirm the practical significance of words: the transition to MASLD was carried out specifically to place the metabolic mechanism at the terminological center; tissue-agnostic oncology became a clinically legitimate model of centering mechanism over organ. Thus, our claim is not that “the term changes everything” but rather the cautious conclusion that “terminology is not insignificant for institutions and clinical reasoning.” At the same time, the NAFLD → MAFLD → MASLD experience also provides an important warning: if terminological reform is pursued too rapidly or without sufficient consensus (73), it may itself generate new fragmentation. It is precisely for this reason that the present article proposes not an immediate replacement of the ICD structure but the addition of a supplementary conceptual layer to the existing system.

The fourth counterargument: neuroprotection is now making a comeback; therefore the organ-centered approach cannot be deemed unsuccessful (57, 58). This objection deserves serious consideration in today’s context. Indeed, positive signals have emerged in recent years: the STAIR XIII (60, 61) document updated cerebroprotection trial priorities, emphasizing alignment of hyperacute protection with reperfusion strategies. The 2025 individual patient data meta-analysis of nerinetide showed clinical benefit signals for certain outcomes. In the LAIS phase III trial, the proportion with excellent outcome was reported to be higher with loberamisal than with placebo. The TASTE-SL trial with edaravone-dexborneol also showed positive results in certain populations (72). Thus, the field cannot be said to have completely “closed.” But it is precisely this comeback that does not weaken our argument; rather, it confirms it in a more nuanced form. Because the field itself is now shifting from the language of “saving the neuron alone” toward the broader language of cerebroprotection, the neurovascular unit, and systemic biology. If success is returning, it is returning more within an expanded, vascular and systemic model. Therefore, the response to this counterargument is not “neuroprotection is wrong” but rather “the field is approaching success through a broader, multi-component framework.” The hemovascular paradigm attempts to express precisely this evolution at the terminological and conceptual level.

The fifth counterargument: new terminology disrupts epidemiological continuity. This is one of the strongest practical objections. Changing terms can affect time series, burden reports, and registries. For this reason, the “hemovascular” paradigm must not disrupt existing statistical flows. It is precisely for this reason that the proposal in this article is formulated not as a replacement coding system but as a supplementary overarching framework. It does not abolish “stroke,” “TIA,” “vascular dementia,” or other phenotypic entities; it only adds a common etiological layer above them. Codes and phenotypic diagnoses are preserved; only the generalizing conceptual language changes. For example, formulations such as “ischemic stroke -- hemovascular cerebral phenotype” or “vascular dementia—hemovascular cognitive phenotype” maintain phenotypic precision while making the etiological center visible. In concrete informatics terms, such a formulation need not require any new primary code: it maps naturally onto the post-coordination mechanism already available in ICD-11, in which a stem code (the organ phenotype, e.g., 8B11 ischaemic stroke) is combined with extension codes that specify the etiological dimension. In a SNOMED CT context, the same logic is expressed through the existing multi-axial structure, where a clinical finding can be related to an underlying etiology through defined relationship attributes rather than a new concept. We therefore envision the hemovascular layer not as an overarching new parent class that competes with existing hierarchies, but as a diagnostic specifier or qualifier applied on top of the preserved organ-phenotype code. This distinction matters: a specifier is additive and back-compatible, leaving all existing single-axis time series intact, whereas a new parent tier would risk exactly the fragmentation the proposal seeks to avoid. Such an approach does not disrupt the flow of historical data but provides scientific and clinical reasoning with a new center. Here, the MASLD experience again provides an important lesson: if a name change is not accompanied by consensus, definitional clarity, and practical integration, it may generate new confusion. For this reason, the hemovascular paradigm is proposed not as a normative call to “abolish the old name everywhere starting tomorrow” but as a conceptual supplement that can be applied incrementally.

It should also be noted that for major international organizations such as WHO, terminological stability is an important value. Institutional conservatism often serves to ensure consistency at the global level. For this reason, the hemovascular paradigm does not reject this institutional reality; it aims to operate more at the level of scientific reasoning and clinical conceptualization.

Overall, these counterarguments indicate that the hemovascular paradigm is not a weak but rather a proposal that must be applied with care. No claim is made that the term “cerebrovascular” must be entirely abolished; the achievements of ICD-11 are not denied; new successes in neuroprotection are not dismissed; epidemiological continuity is not put at risk. This paradigm, while preserving existing achievements, seeks to bring forward the etiological center that remains invisible beneath organ-centered language. It is at its strongest when understood not as a radical replacement but as a conceptual recentering.

### 5.2 The enduring operational value of organ-localization

A balanced treatment of this proposal must acknowledge that organ-centered terminology is not merely a historical artifact; it serves an indispensable operational function, particularly in acute care. In the hyperacute setting, an immediate anatomical label is precisely what mobilizes the correct infrastructure: a “stroke code” activates brain imaging, the thrombolysis pathway, and the neurointerventional team, whereas a “STEMI code” activates the catheterization laboratory and the cardiology team. The deliberately organ-anchored framing of “brain attack” was itself introduced to compress time-to-treatment by conveying urgency through a familiar anatomical analogy. In these contexts, localization is not a cognitive distortion but a triage necessity: the organ of manifestation determines which time-critical, organ-specific intervention must be delivered within minutes.

This observation does not weaken the hemovascular proposal; it delimits its scope. The two framings operate on different timescales and serve different goals. Organ-localization is optimal for hyperacute, phenotype-specific intervention, where the immediate clinical question is “which organ is failing now, and what must be done in the next minutes?” The hemovascular lens is optimal for chronic prevention, risk stratification, multidisciplinary coordination, and research design, where the relevant question is “what systemic process produced this event, and how is the same process threatening other vascular beds?” Far from being mutually exclusive, the two are complementary layers of a single clinical reality: the organ phenotype names the emergency, while the hemovascular spectrum names the disease. Recognizing this division of labor is essential, because it clarifies that the proposal does not seek to remove the operational scaffolding of acute medicine but to add the systemic context that acute scaffolding, by design, sets aside.

### 5.3 From paradigm to practice: education and clinical reasoning

A reasonable question is how clinicians might begin to apply hemovascular thinking now, without waiting for any change to formal classification. Several avenues are already available. In medical education, stroke can be taught not as an isolated neurological emergency but as the cerebral presentation of a systemic vascular process, explicitly linked alongside coronary, renal, and peripheral manifestations within a shared pathobiological narrative; this reframing requires no curricular code change, only a shift of emphasis. In clinical reasoning, the lens translates into concrete habits: routinely screening a patient presenting with one vascular phenotype for disease in other vascular beds, foregrounding modifiable systemic drivers (lipoprotein burden, inflammation, rheological and hemodynamic factors) in prevention planning, and coordinating care across neurology, cardiology, angiology, and nephrology around the shared etiological core rather than in organ silos. At the level of health information systems, as outlined in Section 5.1, the etiological dimension can be recorded as an additive specifier on the existing organ-phenotype code, allowing institutions to begin capturing hemovascular context for analytics and prevention without disrupting established coding workflows. Taken together, these steps illustrate that the paradigm is not contingent on top-down nomenclature reform: its primary near-term value lies in reshaping how clinicians and educators conceptualize, teach, and coordinate vascular care.

### 5.4 Limitations

This article is presented as a conceptual and analytical review. Although the proposed cerebrocentric bias model is built upon historical, linguistic, pathobiological, and translational evidence, it remains a theoretical construct that has not yet been subjected to direct empirical testing. The article establishes that terminological framing can influence clinical reasoning, but it does not yet demonstrate at the experimental level the precise degree to which “cerebrovascular” and its alternative formulations affect physician decisions or diagnostic priorities. For this reason, the most important test of this model at the next stage should be conducted through randomized vignette studies and simulated clinical scenarios—designs that permit direct assessment of which diagnostic pathway physicians choose when the same clinical case is presented within different terminological frames.

The second important limitation is that explaining translational failures in stroke solely through terminology would be an oversimplification. Pharmacological limitations, time windows, patient heterogeneity, and methodological problems played significant roles in the history of neuroprotective trial failures. For this reason, this article interprets cerebrocentric bias not as the sole cause but as a conceptual factor that may have contributed to which layer received greater scientific attention. This caution is particularly important in today’s context, as new positive signals in neuroprotection and the broader cerebroprotection direction are emerging within the field. This suggests that the field was not entirely on the wrong path but is evolutionarily transitioning toward a broader, multi-component model.

The third limitation is that any mechanism-based reclassification has its own boundaries. Tissue-agnostic oncology powerfully demonstrated this possibility, yet the same molecular marker does not yield identical results across different organs. For example, BRAF-targeted therapy may be effective in melanoma, but the response in colorectal cancer is constrained by an entirely different biological context. Mechanism-based classification therefore does not completely override organ context. Similarly, the hemovascular paradigm should be understood not as an approach that eliminates organ phenotypes but as one that repositions them under a common etiological framework.

The fourth limitation is that terminological reform itself is never a sufficient condition. Changing a name does not change a biological process and does not automatically introduce new guidelines. Nevertheless, in certain historical instances, terminological recentering has served as a necessary condition: it opened a linguistic doorway for seeing the disease differently and formulating new priorities. The claim of this article is precisely this: the hemovascular paradigm alone does not solve the problem, but it may be a necessary conceptual step for making the pathogenetic center visible again in a context where organ-centered misalignment persists. The authors’ ongoing experimental studies (28) are aimed at testing the pathogenetic model of the hemovascular paradigm experimentally.

The fifth limitation relates to institutional implementation. Even when nomenclature reforms at the WHO level are scientifically well-founded, their implementation process is by nature cautious, multi-staged, and consensus-based. For this reason, even if the hemovascular paradigm is theoretically strong, its transfer to the level of formal classification is likely to be not rapid but evolutionary and incremental. This article is not a direct call for nomenclature replacement but should be viewed as a theoretical foundation for future empirical testing, consensus processes, and classification discussions.

## 6 Conclusion

This article revisited the relationship between terminology, clinical reasoning, and pathogenetic hierarchy using stroke as a case study. The analysis was conducted at the intersection of psycholinguistics, cognitive bias research, translational stroke science, pan-vascular medicine, and modern multi-organ disease models. Three main conclusions emerged: terminological, structural, and conceptual. Their collective meaning is that while the organ-centered language currently used to describe stroke does not entirely misrepresent the biological process, it does not fully reflect its pathogenetic hierarchy.

First, the terminological conclusion is that organ-centered naming generates the same systemic problem at two levels. At the macro level, the term “cardiovascular” places the heart at the semantic and institutional center, casting stroke into the shadow within the broader spectrum. At the micro level, the term “cerebrovascular” foregrounds the brain, relegating the pathogenetic root—blood composition changes, systemic arterial pathology, and hemodynamic mediation—conceptually to the background. In both cases, the consequence occupies the center of the term while the cause remains at its margins. This semantic inertia may indirectly influence clinical reasoning, research priorities, and institutionalized service structures. The translational failure history of 1,026 experimental neuroprotective agents and the subsequent shift of the field toward broader cerebroprotection and the neurovascular unit is also consistent with the probable cumulative consequences of this organ-centered thinking.

Second, the structural conclusion is that this terminological misalignment does not remain solely at the level of language; it also intersects with classification architecture. The problem of fragmentation of the cerebrovascular spectrum within the ICD exists and has been examined in detail in a separate analytical article (28). Within the scope of the present article, the key point is that the terminological center and the classification center do not always align with the pathogenetic center. While ICD-11 has introduced important corrections, it has not fully recentered the historical organ-phenotypic language. Terminological and classificatory levels are therefore not separate from each other; they draw from the same conceptual root.

Third, the conceptual conclusion is that the hemovascular paradigm offers an alternative framework more closely aligned with the pathogenetic hierarchy. This paradigm views the disease through a three-stage model: the primary stage—blood composition changes and systemic arterial pathology; the secondary stage—hemodynamic disturbances; the tertiary stage---multi-organ clinical phenotypes. Under this approach, stroke is understood not as an isolated event but as the cerebral phenotype of the hemovascular spectrum. This model is concordant with the emergence of pan-vascular medicine, clinical achievements in the COMPASS and CANTOS trials that targeted the etiological center, and the appearance of multi-system clinical platforms such as the CKM syndrome.

At the same time, this article does not advance a radical or revolutionary claim. The hemovascular paradigm does not propose to abolish organ phenotypes; it preserves clinical entities such as “stroke,” “TIA,” “myocardial infarction,” and “vascular dementia” while adding a higher-order etiologically unifying framework above them. The essence of this approach is therefore not replacement but recentering. For this reason, the article’s final position is evolutionary in character: not the rejection of existing clinical language and classification practice but rather their approximation to the current level of pathobiological knowledge. It is precisely for this reason that the hemovascular paradigm should be viewed not as a revolutionary but as an evolutionary proposal.

## Data Availability

The original contributions presented in the study are included in the article/supplementary material, further inquiries can be directed to the corresponding author.
